# Emergency Management of the Prevention and Control of Novel Coronavirus Pneumonia in Specialized Branches of Hospital

**DOI:** 10.1111/acem.13958

**Published:** 2020-03-23

**Authors:** Xiuqing Ma, Shiyu Li, Shaobin Yu, Ying Ouyang, Lin Zeng, Xiao Li, Hai Li

**Affiliations:** ^1^ West China Hospital Sichuan University Chengdu China

In December 2019, an epidemic of novel coronavirus pneumonia (NCP) broke out in Wuhan, Hubei Province. The outbreak was severe and coincided with the Spring Festival travel season. On January 15, 2020, the West China Hospital of Sichuan University, a large hospital in China, held a seminar on prevention and control in accordance with the requirements of the National Health Commission on Prevention and Control. On January 16, the emergency plan for the prevention and treatment of NCP in West China Hospital of Sichuan University was formulated for the first time. The president of the university was named secretary of the “Respiratory Infectious Disease Prevention and Control Leading Group” and medical treatment expert group. Wenjiang District Hospital of West China Hospital, a branch of West China Hospital of Sichuan University, is located in Wenjiang district of Chengdu, 23 kilometers away from the main hospital district. It mainly focuses on specialties, such as rehabilitative medicine, lung cancer, and sports medicine. It does not have a separate emergency department and fever clinic but implements integrated and unified management with the main hospital area. In the face of such an unusual and unpredictable epidemic, how to ensure smooth government order, effective measures, and prevention and control to prevent outbreaks in the subdivision area of hospitals is a new test for specialized subdivision areas of hospitals. The following sections present the emergency management experience of Wenjiang Hospital in West China in the prevention and control of the NCP epidemic.

## SET UP A LEADING GROUP FOR EPIDEMIC PREVENTION AND CONTROL

Faced with the severity of the Wuhan epidemic situation, all departments and personnel attach great importance to positive response, and taking effective epidemic prevention measures is particularly important. As a subhospital of West China Hospital, Wenjiang Hospital maintains the same level of care as the main hospital at all times. According to the actual situation and architectural characteristics of the hospital, prevention and control measures are rapidly deployed according to the local conditions to prepare for the outbreak before it arrives. According to the overall requirements of the hospital, under the organization and leadership of the director of the subdivision hospital, the NCP epidemic prevention and control working group of the branch was established. The head of the subdivision hospital serves as the group leader; the chief of the medical section, the infection control administrator, and the director of the outpatient department serve as the deputy group leaders; and the heads of the respiratory specialty, ICU, lung cancer center, laboratory, equipment department, logistics, security, radiation, color Doppler ultrasound, cleaning, and other related departments serve as team members. All members work together; closely monitor the development of the epidemic situation in the whole country, hospitals, and hospital areas; report the epidemic situation to relevant departments in an accurate and timely fashion; and organize regular meetings to discuss, formulate, and revise prevention and control plans and measures and are responsible for the full implementation of plans, resource organization, and coordination.

## MAKE CONTINGENCY PLANS

According to the report, NCP is highly contagious, and most of the deaths are from heart, lung, and other chronic underlying diseases.^1^ Due to the Spring Festival, the number of patients in the hospital is <40, but they cannot be discharged because of the relatively serious basic diseases. Once outpatient clinics are opened, patients from all over the country will become vulnerable to nosocomial infection of NCP. In accordance with the technical guidelines for the prevention and control of NCP in medical institutions (first edition), suspected cases of fever must be isolated in place.^2^ To minimize cross‐infection in the hospital caused by NCP. On January 17, 2020, the NCP epidemic prevention and control working group of Wenjiang District carefully studied the architectural features and space utilization of the district, formulated an emergency plan for the prevention and control of NCP in the affiliated district, and set up a preexamination and triage station and a tent observation area for patients with fever at the entrance of the hospital. The isolation observation room was rebuilt according to the relevant provisions of the “technical code for hospital isolation.”^3^ The objective is to isolate patients with fever or suspected cases for early detection and minimize cross‐infection in the hospital. The NCP emergency plan regarding guidelines for prevention and control in subhospital areas will be regularly adjusted and improved according to the provisions and control requirements of the country and the hospital in different periods.

## STRENGTHEN PERSONNEL EDUCATION AND TRAINING

The outbreak of the NCP in Wuhan has much to do with people's early cognition of and attention to the epidemic and the lack of epidemic prevention knowledge; from organizations to individuals and from professionals to ordinary people, there is a lack of knowledge to different degrees. Therefore, it is particularly important to prevent and control the epidemic in subhospital areas and strengthen education and training for all kinds of personnel. Wenjiang Hospital in West China intends to strengthen the education and training of staff regarding knowledge, information and regulations related to epidemic prevention and control through WeChat, QQ, TV, video, and other media as well as through listening to important speeches and work arrangements online. For the patients and their family members, they should receive education and training on epidemic prevention knowledge and information about special management requirements of the hospital during the epidemic through small unit modes such as bedside or nursing groups as well as through television, posters, and admission propaganda and education communication. Through multiple approaches, employees, patients, and family members can pay more attention to and understand the epidemic situation; gain enhanced awareness of prevention and control; master the right protection skills; and reduce the chance of cross‐infection.

## ORGANIZE AND COORDINATE RESOURCES

For the prevention and control of any outbreak, personnel and material organization and preparation are necessary. As a noninfectious hospital, the hospital's stock base of protective materials for outpatients without fever is limited. Wenjiang Hospital in West China immediately mobilized reserves for major epidemic prevention and control. First, all the protective equipment in stock is sorted and listed. Supplies are mobilized and reserved based on the estimated safety stock and minimum basic requirements for the area of operations that may be involved in protection. The materials in each department are under special management according to the postclassification accounting, and quantitative distribution, zero inventory, real‐time distribution at the initiative of the whole hospital, and saving are advocated. Important and scarce materials, such as goggles and medical isolation clothing, should be rationed reasonably according to the three‐level protection guide by the infection control administrator, and the dynamic balance of protection materials should be regularly monitored to ensure necessary protection and avoid waste. Emergency rescue teams and volunteer service teams should be established to achieve the required manpower for prevention and control work.

## IMPLEMENT A THREE‐LEVEL PREVENTION AND CONTROL MECHANISM

To ensure the effective implementation of NCP prevention and control measures and to effectively and resolutely control the occurrence of NCP, a three‐level prevention and control monitoring system was adopted in Wenjiang Hospital in West China. Primary prevention and control monitoring are set up at the gate of the hospital, secondary monitoring is set up at the only entrance of the building, and tertiary monitoring is set up at each reception point and ward in the building. At each gate, professional nurses and security personnel are responsible for strict temperature monitoring, personnel identification, source inquiries, and registration of the personnel coming in and going out. The key questions are whether people entering the hospital come from Wuhan or other key epidemic areas, key patients are interrogated and closely monitored, and suspected patients are observed in time for timely isolation and treatment. To further ensure the effective implementation of various measures, the subdivision hospital also adopted a three‐level supervision mechanism. The first level is the director, who conducts site inspection once a week and carries out in‐depth supervision of the effectiveness of epidemic prevention in hospital areas, understands the daily epidemic situation and work situation, and then provides timely guidance and coordination in case of difficulty or doubt. The second level is the supervision of the department of infection control. Each unit is inspected at least three times a week to check whether the protective measures of various departments and personnel are in place and effective. The third level is the daily routine inspection of monitoring and protection of each unit of the hospital by the head nurse. The head nurse supervises the key monitoring links in the first and second levels to ensure the effective implementation of the measures in each link. In addition, the hospital accepts the supervision and inspection of local health law enforcement departments, thus forming the joint prevention and control of multiple departments.

## ENVIRONMENT AND ACCESS MANAGEMENT

The outbreak is fierce and insidious, spread mainly by droplets and contact^4^ and clearly can be transmitted from person to person.^5^ To completely cut off the source of infection, the country urgently took decisive measures to seal off cities in some areas, and various provinces and cities have issued a variety of bans and closed public places, such as tourism sites, restaurants, and cinemas.^6^ On February 18, 2020, the diagnosis and treatment program for new coronavirus pneumonia (trial version 6) made it clear that asymptomatic infected people could also become a source of infection, and there was a possibility of aerosol transmission under the condition of prolonged exposure and high concentration of aerosol in a relatively closed environment.^7^ Therefore, in accordance with relevant regulations, a series of more stringent control measures have been adopted. To avoid crowd gathering and reduce the close contact between people, the subdivision hospital and departments strictly implement the management of three channels. The patient channel is completely separated from the staff. Patients enter and exit the hospital following one way, and the staff enter and exit through another channel. Given that the main building of the hospital has only four floors, the elevators are divided into patient elevators and logistics elevators, and the employee elevator is no longer in use to encourage employees to use the stairs. All other channels are closed. Each channel has clear and eye‐catching signs for guidance; additional security, nursing personnel, volunteers, and other specially assigned staff are assigned supervision and guidance roles; and crowds are dispersed 1 meter apart to avoid contamination and close contact. Moreover, hospital fire inspection should be strengthened to ensure a special control period of fire safety. Canteen service is canceled, and delivery service is provided^8^ to enhance the cleanliness and disinfection of the hospital floor and surface of objects. According to regulations for strengthening the management of medical waste of NCP, special routes are planned to strengthen the disinfection of sewage, and the frequency of testing is increased from quarterly to monthly. The cleaning, disinfection, and operation modes of the air‐conditioning ventilation system are in line with the requirements of the “cleaning and disinfection code for centralized air‐conditioning and ventilation systems in public places” (WS/T 396‐2012). The operation mode is changed to the fresh air mode, the return air system is closed, the fresh air volume is increased, and the windows are frequently opened for ventilation.^9^, ^10^

## WARD AND BUSINESS MANAGEMENT

During the epidemic prevention and control period, access control management was strictly enforced in the ward. Each unit was separated by access control or a temporary fence, forming a relatively independent and safe area. The entrance and exit were carefully managed and guarded by professional nurses, and access was restricted. Every person entering the ward must verify his/her identity as an employee, patient or visitor, have his/her temperature monitored, and wear a mask, and the residence history of accompanying visitors was carefully checked. All personnel were required to refrain from unnecessary medical activities and prohibited from walking to other places. To reduce crowd gathering, the number of companions was limited to one per patient, and whether visitors were allowed to stay depended on the condition of the patient. Each patient admitted to the hospital was required to fill out the “outbreak‐related investigation form” and signed the “informed consent form for accompanying management” and “epidemic commitment form.” The management of all staff in medical care, work, property, administration, etc., was strengthened; those who had an epidemiologic history, fever, or other discomfort were strictly isolated, and daily monitoring and reporting were performed. Ophthalmology, otolaryngology, physical examination, endoscopy, etc., were stopped. The number of outpatients and inpatients was reduced, and all departments were to be gradually opened to the public according to the epidemic situation.^8^ The admission and treatment of patients were carried out according to priority procedures, such as critical illness and restrictive surgery.

The outbreak of the NCP was so severe that it quickly broke out in Wuhan and spread across the country, making prevention and control extremely difficult. As of 12 pm on February 27, 2020, a total of 31 provinces (autonomous regions and municipalities directly under the central government) and the Xinjiang production and construction corps had reported 1,770 deaths and 78,824 confirmed cases.^11^ The state took national efforts to protect Wuhan, Hubei Province; many cities in Hubei Province were closed off; and more than 30,000 medical personnel were mobilized from all over Hubei Province to help. The situation is extremely serious. The specialized branch area of West China Hospital of Sichuan University, under the leadership of the local government, attaches great importance to the subhospital director to lead the whole team; achieve quick, sustained, and orderly response; achieve effective organization; achieve full mobilization of resources and epidemic prevention; play the role of gatekeeper; and protect the general staff and the patients' lives. From January 25 to February 27 (during the epidemic prevention and control period), a total of 8,863 outpatient patients, 3,874 hemodialysis patients, 3,400 inpatients, 119 surgeries, and 10 fever patients were protected from NCP, and no NCP occurred in the hospital. The control of epidemic prevention in the subdivision area was effective. Of course, all epidemic prevention measures are not immutable and need to be revised and adjusted according to the development of the epidemic situation and gradually improved. The only constant is the high and close attention to the epidemic situation. Effective emergency plans should be formulated from the very beginning, and safety control measures should be taken decisively. Only in this way can the infection be controlled to the minimum.

The authors thank all the participants and the nursing team of West China Hospital of Sichuan University who helped to collect and organize the data.
